# 上皮间质转化与肿瘤耐药

**DOI:** 10.3779/j.issn.1009-3419.2013.01.10

**Published:** 2013-01-20

**Authors:** 志浩 吴

**Affiliations:** 1 300052 天津，天津市肺癌转移与肿瘤微环境重点实验室，天津市肺癌研究所，天津医科大学总医院 Tianjin Key Laboratory of Lung Cancer Metastasis and Tumor Microenvironment, Tianjin Lung Cancer Institute, Tianjin Medical University General Hospital, Tianjin 300052, China; 2 300052 天津，天津医科大学总医院肿瘤科 Department of Oncology, Tianjin Medical University General Hospital, Tianjin 300052, China

**Keywords:** 上皮间质转化, 肿瘤, 耐药, Epithelial-mesenchymal transition, Tumor, Drug resistance

## Abstract

肿瘤药物治疗过程中常常要面临肿瘤细胞耐药的问题。上皮间质转化（epithelial-mesenchymal transition, EMT）在肿瘤耐药方面的作用为解决该问题提供了可能。该文围绕EMT基本特征、EMT与肿瘤耐药的关系、EMT在肿瘤耐药过程中机制的研究进展进行详细综述。

## Abstract (en)

Resistance to antineoplastic drugs is a common problem in cancer treatments. Epithelial-mesenchymal transition (EMT), which plays an important role in the process of drug resistance, may provide opportunity to solve this problem. This article reviews the characteristics of EMT, relationship between EMT and drug resistance, mechanism of EMT in tumor drug resistance in details.

肿瘤细胞对抗肿瘤药物产生耐药是导致治疗失败的常见原因。然而，目前研究尚未发现确切的能逆转肿瘤耐药的有效途径，肿瘤耐药仍是困扰肿瘤治疗的关键性难题。近期研究发现，肿瘤耐药与肿瘤细胞发生上皮间质转化（epithelial-mesenchymal transition, EMT）密切相关，EMT在抗肿瘤治疗耐药中的作用受到越来越多的关注^[1]^。

## 1 EMT的概念与特征

EMT是指上皮细胞通过特定程序转化为具有间质表型细胞的生物学过程。EMT是Greenberg和Hay^[2]^在1982年提出的，他们发现晶状体上皮细胞在胶原凝胶中可以形成伪足，转变为间质细胞样的形态。此后，陆续有报道^[3]^发现很多物种的原肠胚、心瓣膜、肌肉、骨骼系统的形成都有赖于EMT。近期研究^[1, 4]^发现，EMT现象与细胞的生物学行为密切相关。目前认为，EMT是由微环境因子作用于细胞受体，诱导细胞信号传导通路发生变化，最终导致基因表达改变而形成的^[4]^。

综合国内外有关EMT基本特征的报道，总结如下：①细胞形态的变化：发生EMT的上皮细胞丧失细胞极性，变成长梭形、分散排列的间质细胞; ②细胞表面标志物变化：发生EMT的细胞上皮细胞标志蛋白如E-钙黏蛋白（E-cadherin）和角蛋白等表达下调，间质细胞标志蛋白如波形蛋白（Vimentin）、纤维连接蛋白（Fibronectin）、神经钙粘蛋白、甲平滑肌抗原-SMA等表达上调; ③细胞生物学行为变化：发生EMT的上皮细胞由静止状态变为运动、迁移能力较强的间质细胞，相关蛋白质溶解酶可降解基膜使细胞侵入细胞外基质中。并非经历EMT的细胞均具备以上三个变化，但细胞迁移能力的获得是细胞EMT的重要标志^[5, 6]^。

## 2 肿瘤细胞EMT现象

人体的恶性肿瘤中绝大多数是上皮细胞性肿瘤，上皮肿瘤细胞发生EMT与肿瘤细胞侵袭转移、肿瘤干细胞形成以及耐药等行为均有着密切关系。首先，EMT是上皮细胞获得迁移能力的方式，因而它成为上皮细胞癌浸润、转移的重要途径，越来越多的研究^[7]^表明它与上皮细胞恶性肿瘤的侵润和转移关系密切。原发上皮肿瘤边缘处发生局部侵润的细胞常失去细胞极性以及与基底膜连接等上皮细胞表型，获得具有较高的迁移、侵袭和降解细胞外基质能力的间质细胞表型，这是肿瘤离开基底膜侵润和转移到正常组织的必备条件，同时EMT后细胞穿透血管内皮细胞进入血液循环的能力也增强，这些都显示EMT与肿瘤侵袭和转移的早期阶段密切相关^[5, 8]^。其次，EMT与肿瘤干细胞表型的形成、转化过程有着密切的联系。发生EMT的肿瘤细胞往往具有干细胞特征，这些细胞常高表达CD44^high^、CD24^low^或者CD133^+^亚群。研究^[9, 10]^发现，EMT和肿瘤干细胞形成的过程存在相同的调控因子和信号通路（例如TGF-β），组织上皮细胞可能通过EMT过程形成肿瘤干细胞，从而调控肿瘤的形成和分化。除此之外，EMT在肿瘤细胞耐药过程中也起到非常关键作用。

## 3 EMT与肿瘤耐药

肿瘤细胞在产生获得性耐药的过程中有间质化的趋势，而本身具有间质分化状态的肿瘤细胞也常表现为原发性耐药的特点，EMT已逐渐被认为是肿瘤耐药的一个新的重要机制^[4]^。

最初研究^[11]^发现耐奥沙利铂的结肠癌细胞株呈现长梭形、极性丧失、细胞分离、伪足形成等特征，免疫荧光检测发现该细胞株的上皮标志蛋白E-cadherin表达下调、间质标志蛋白Vimentin表达上调。随后研究^[12]^又在耐紫杉醇的卵巢癌细胞株中发现，EMT相关转录因子Snail、Twist表达上调，侵袭相关的蛋白质溶解酶如基质金属蛋白酶-2和膜型-1-基质金属蛋白酶均增加，致瘤实验显示腹膜下注射耐药细胞株的小鼠形成腹膜播散转移较紫杉醇敏感组小鼠多。此后的研究还发现阿霉素体外可诱导乳腺癌细胞发生EMT，EMT相关转录因子Twist表达增多，且只有发生EMT的细胞才表现出侵袭转移能力增强和多药耐药（multi-drug resistance, MDR）现象^[13]^。更多的研究在耐他莫昔芬的乳腺癌细胞株^[14]^、耐吉西他滨的胰腺癌细胞株^[15, 16]^、耐顺铂的胰腺癌细胞株^[16]^以及耐5-Fu的乳腺癌、结肠癌、胰腺癌细胞株中均观察到了EMT现象^[17, 18]^。

分子靶向药物的耐药也与EMT关系密切。目前认为表皮生长因子络氨酸激酶抑制剂（epidermal growth factor receptor tyrosine kinase inhibitors, EGFR-TKIs）获得性耐药的机制主要包括T790M二次突变和酪氨酸激酶受体家族基因*MET*扩增等，然而，临床研究发现既没有二次突变也没有*MET*基因扩增的EGFR-TKIs耐药患者肿瘤组织细胞呈现间质特征^[19]^。厄洛替尼耐药的非小细胞肺癌细胞株H460和Calu6上皮细胞标志蛋白E-cadherin表达降低，而厄洛替尼敏感细胞株H292的E-cadherin则呈现高表达^[20, 21]^。吉非替尼耐药的肺癌细胞株H157、H1730、A549、H520上皮细胞标志蛋白E-cadherin、上皮细胞粘附分子EpCAM、紧密连接体蛋白claudin4和caludin7的表达普遍较低，而吉非替尼敏感细胞株H322、H358、H1648这些蛋白表达则增高^[22-25]^。相反，吉非替尼耐药细胞株间质细胞标志蛋白Vimentin增高，而敏感细胞株中这种蛋白的表达则降低^[22-25]^。

## 4 EMT在抗肿瘤药物耐药中的作用机制

越来越多的研究表明，EMT与肿瘤细胞耐药存在共同的信号调控途径，EMT相关信号传导通路的激活和失调在肿瘤细胞间质化过程以及抗肿瘤药物耐药的过程中起协同的调控作用。现有研究^[26-31]^表明，调控肿瘤细胞间质化状态的信号途径包括PI3K/Akt/GSK-3β/Snail、NF-κB/Snail/YY1/RKIP/PTEN环路、PI3K/Akt/HIF-1α、Notch-2、JNK/线粒体、MAPK/线粒体相关通路都已被证明可通过EMT在顺铂、吉西他滨、吉非替尼等抗肿瘤药物耐药过程中起关键性作用。

在EMT相关信号通路的调控作用下，EMT相关转录因子（Zeb1、Slug）在抗肿瘤药物耐药中起关键调控作用，抑制EMT相关转录因子活性，阻止其调控下游因子的能力，可以逆转耐药细胞间质化状态，实现恢复对抗肿瘤治疗的敏感性。研究发现，EMT相关转录因子Zeb1可以通过恢复组蛋白去乙酰化的作用抑制E-cadherin表达促使细胞间质化，组蛋白去乙酰抑制剂MS-275则能抑制这种作用而增加E-cadherin表达，使间质化的耐药细胞恢复上皮细胞特征，从而逆转耐药细胞EMT状态，该作用已在逆转吉非替尼、吉西他滨耐药的Ⅰ期临床试验中得到证实^[23]^。此外，EMT相关转录因子Slug在吉非替尼获得性耐药的过程中也起到关键作用，靶向敲除Slug表达能逆转耐药细胞株的间质化特征，恢复耐药细胞株对吉非替尼的敏感性^[32]^。

肿瘤细胞MDR过程中也伴随着EMT的发生，高表达P-gp的多药耐药肿瘤细胞常表现出较强的侵袭、转移能力^[33]^。研究发现，PI3K/Akt、MAPK等信号通路的激活能上调P-gp的表达，从而导致肿瘤MDR的发生^[34]^，而PI3K/Akt、MAPK等信号通路是EMT过程中重要的调控途径^[35-37]^。这说明EMT和MDR的发生过程中共同的信号通路在两者的发生机制中可能起到某种作用。此外，研究表明P-gp蛋白以及在MDR过程中起重要作用的ATP结合级联转运蛋白均受EMT相关转录因子Snail、Twist的调控，这些都进一步说明了EMT与MDR之间可能存在共同的调控机制^[38-40]^。

## 5 问题与展望

综上所述，EMT在肿瘤细胞耐药中的作用为解决抗肿瘤药物耐药的难题提供了可能，但仍存在许多争议之处和亟待解决的问题。例如恶性肿瘤组织标本中EMT检测新方法的探索、肿瘤细胞EMT调控耐药的多种机制和信号通路信息网络的完善、以EMT信号通路作为靶点逆转耐药应用于临床能否能达到预期的治疗效果等。因此，以EMT为切入点研究恶性肿瘤的耐药还存在广阔的研究空间和应用前景。
